# Impact of climate, rising atmospheric carbon dioxide, and other environmental factors on water-use efficiency at multiple land cover types

**DOI:** 10.1038/s41598-020-68472-7

**Published:** 2020-07-15

**Authors:** Muhammad Umair, Daeun Kim, Minha Choi

**Affiliations:** 1 Environment and Remote Sensing Laboratory, Department of Water Resources, Graduate School of Water Resources, Sungkyunkwan University, Suwon, 16419 Republic of Korea; 2 Center for Built Environment, Sungkyunkwan University, Suwon, 16419 Republic of Korea

**Keywords:** Carbon cycle, Climate change

## Abstract

Rising atmospheric CO_2_, changing climate, and other environmental factors such as nitrogen deposition and aerosol concentration influence carbon and water fluxes significantly. Water-use efficiency (WUE) was used to analyze these factors over 3 decades (1981–2010) using the Community Land Model 5.0 (CLM5.0). The study analyzes the effects of climate and other environmental factors on multiple land cover types (forest, grassland, and cropland) with divided study periods (1981–2000 and 2001–2010). Ecosystem WUE (EWUE) and transpiration WUE (TWUE) increased at the forest site due to the CO_2_ fertilization effect but decreased at the grassland and cropland sites due to lower gross primary production and higher/lower (cropland/grassland) evapotranspiration as consequences of rising temperature and water availability. Inherent WUE confirmed that EWUE and TWUE trends were controlled by the rising temperature and CO_2_-induced warming through an increase in vapor pressure deficit. In this way, forest and cropland sites showed warming patterns, while the grassland site showed a drier climate. The later period (2001–2010) showed steeper trends in WUE compared with the earlier period at all sites, implying a change in climate. The results showed implications for rising temperature due to increased CO_2_ concentration at multiple land cover types.

## Introduction

Water-use efficiency (WUE) is a critical component that links the carbon and water cycles through the relationship between gross primary production (GPP) and evapotranspiration (ET)^1–3^. The WUE characterizes the coupling between terrestrial ecosystems and atmospheric components such as GPP and ET at various land cover types (LCTs) (e.g., forest, grassland, and cropland) and is essential to understanding climate variability^4–7^. Each LCTs affect the carbon and water cycles differently, the WUE can be dynamic^1,8,9^ because it depends upon roughness length, albedo, solar irradiance, precipitation, and especially the coupling between GPP and ET^10–13^. WUE is not only modulated by changes in climatic factors such as precipitation, solar radiation, air temperature, wind speed, humidity, and atmospheric pressure, but also by environmental factors such as increase in CO_2_, nitrogen deposition, and aerosol concentration^14–16^.

Among the environmental factors, increase in atmospheric CO_2_ has especially a dramatic effect on WUE; according to the Intergovernmental Panel on Climate Change (IPCC–2018), CO_2_ has been increasing at an annual rate of 1.8 ppm year^−1^ over the last 4 decades. CO_2_ concentration in the atmosphere can influence WUE in two ways. First, in structural terms, rising CO_2_ positively affects the photosynthesis process, stimulating carbon assimilation, and decreasing stomatal conductance, which increases the leaf area index (LAI)^17–19^. In physiological terms, plants control the opening and closing of their stomata in response to prevailing atmospheric CO_2_^2,3,8^. Both processes can increase or decrease the ecosystem WUE (EWUE) depending on the LCTs and soil moisture availability at the surface^15,20^.

This study evaluated EWUE trends and addressed the phenomena of structural and physiological effects of CO_2_ concentration with multiple LCTs and climatic variables at a point scale. According to Keenan et al.^2^, the long-term increasing EWUE trend in forest ecosystems is due to the CO_2_ fertilization effect. Green et al.^13^ described GPP and its relationship with soil moisture, which has a significant influence on carbon uptake at a global scale. Liu et al.^12^ studied WUE and its response to drought in detail in China’s ecosystem from 2000 to 2011 and explained the trends in different regions of the country. Huang et al.^3,15^ discussed multiple definitions of WUE in detail, including seasonal changes according to various land surface models (LSMs) and their impact on climate and on a global scale from 1982 to 2008. However, previous studies have not addressed long-term trends in EWUE, including the effects of various environmental factors, in multiple LCTs. Filling this gap is the main goal of the current study.

In addition to changing climate and increasing CO_2_, WUE can be affected by nitrogen limitation and aerosol concentration in the atmosphere^21,22^. Norby et al.^21^ explained that in 2001–2003, despite an increase in CO_2_ concentration, net primary production (NPP) declined due to nitrogen limitation in forest ecosystems. The aerosol concentration in the atmosphere decreased the amount of solar radiation reaching the land surface, which affected ET and in turn EWUE^23^.

Furthermore, the effects of climate variability and environmental factors on vegetation should also be assessed in detail considering transpiration (T_r_)-based WUE^15^ (TWUE). Another form of WUE, inherent water-use efficiency (IWUE), which is based on vapor pressure deficit (VPD), is critical to evaluate as it changes due to climate warming^24,25^. The VPD is driven by air temperature and negatively affects IWUE due to strong coupling with ET^26^. Previous studies have demonstrated that GPP is sensitive to increasing VPD, as well as soil water deficits, in climate warming conditions that affect IWUE^27–29^.

The scientific questions addressed in this study were: (1) how do multiple environmental factors affect the terrestrial ecosystem (EWUE)? (2) How do climate, CO_2_ level rise, aerosol concentration, and nitrogen deposition affect multiple land cover types? (3) How has the climate changed over the 3 decades between 1981 and 2010? The objectives were designed to address these questions and included the investigation of long-term trends of EWUE, TWUE, and IWUE over the forest, grassland, and cropland sites (CN-Qia, CN-Cng, and US-Ne3, respectively, Table 1) from 1980 to 2010. To achieve this, the Community Land Model (CLM5.0; Lawrence et al. 2018) was applied at the three study sites to quantify the effects of four primary environmental factors. Using CLM5.0, climate change (‘CLIM’), increasing carbon dioxide (‘CO_2_’), nitrogen deposition (‘NDEP’), and aerosol concentration in the atmosphere (‘AERO’) were the four factors considered in this study (Table 2). Actual water and carbon flux data from flux towers were used to validate the performance of CLM5.0. Statistical analysis provided deeper insights into the effects of climatic and environmental factors affecting the water and carbon fluxes.

**Table 1:** Meteorological and field characteristics of the selected study sites.

Site	Location		Study period	Plant functional type	Soil texture	Climate zone	Mean annual temperature (°C)	Mean annual precipitation (mm)
	Country	Latitude/longitude (elevation)			Sand/clay			
Qianyanzhou (CN-Qia)	China	26.74°/115.06° (109 m)	2003–2005	Evergreen Needleleaf Forest (ENF)	52/24 (sandy–clay loam)	Cfa—warm temperate	18.25	1,170
Changling (CN-Cng)	China	44.59°/123.51° (143 m)	06/01/2007–09/30/2010	Grassland (GRA)	89/5 (sand)	Bsk—arid	4.90	400
Mead–rainfed maize-soybean rotation site (US-Ne3)	United States	41.18°/‒ 96.44° (363 m)	2008–2010	Cropland (CRO)	35/41 (loam)	Dfa—boreal	10.11	784

**Table 2:** Experimental design.

Simulations		Driving factors			
		Climate	Increasing CO_2_	Aerosol concentration	Nitrogen deposition
E1	CLIM	Yes	No	No	No
E2	CLIM + CO_2_	Yes	Yes	No	No
E3	CLIM + AERO	Yes	No	Yes	No
E4	CLIM + NDEP	Yes	No	No	Yes
E5	All	Yes	Yes	Yes	Yes

E1 with transient climate, E2 with transient climate and CO_2_, E3 with transient climate and aerosol concentration, E4 with transient climate and nitrogen deposition, and E5 considers all the environmental variables and climate.

## Results

### Validation of CLM5.0 with flux tower data

Figures S1 and S2 show daily time series and scatter plots for ET and GPP, respectively, at the three sites with CLM5.0 and flux tower data. The cropland site (US-Ne3) showed the best results for the estimation of ET, with coefficient of determination (R^2^) and slope of 0.74 and 0.82, respectively (Fig. S1 and Table 3). The other two sites showed the reasonable agreement of model simulations with flux tower observations. The R^2^ and slope were 0.61 and 0.57, respectively, for CN-Qia and 0.59 and 0.84 for CN-Cng. The index of agreement (IA), bias, and root mean square error (RMSE) also showed better results for the cropland site, with values of 0.93, 0.05 mm day^−1^, and 0.79 mm day^−1^, respectively. Model simulations underestimated ET at the forest and grassland site with negative biases of − 0.40 and − 0.12 mm day^−1^ (Figs. S1a and S1b, Table 3), which may be due to forcing data and structural uncertainty of the model (details in the “Potential uncertainties and limitations” section). At the cropland site, underestimation by model simulations were observed during the peak growing season, however in the spring season, the ET was slightly overestimated (0.05 mm day^−1^), possibly due to early onset of the spring season effected by the forcing data uncertainties in the model^30^ (Fig. S1c, Table 3).

**Table 3:** Validation of CLM 5.0 results with FLUXNET observations from three sites at daily time scale.

		R^2^	RMSE	Slope	Bias	IA
CN-Qia (evergreen needleleaf forest)	GPP	0.71	2.08	0.63	– 0.04	0.80
	ER	0.59	2.17	0.26	– 1.67	0.60
	ET	0.61	1.09	0.57	– 0.40	0.73
CN-Cng (grassland)	GPP	0.26	2.55	0.54	0.32	0.64
	ER	0.40	1.86	1.06	0.94	0.67
	ET	0.59	0.74	0.84	– 0.12	0.87
US-Ne3 (cropland)	GPP	0.49	4.48	0.45	0.33	0.79
	ER	0.69	2.17	0.90	1.14	0.87
	ET	0.74	0.79	0.82	0.05	0.93

Units: [GPP, ER (gC m^−2^ day^−1^); ET (mm day^−1^)] for RMSE and Bias.

For GPP estimation using CLM5.0, the forest site (CN-Qia) showed the best results, with R^2^ = 0.71 and a slope of 0.63 (Fig. S2a, Table 3). The R^2^ values and IA showed better results from model simulations with in-situ data for forest and cropland sites than grassland site. The grassland site (Fig. S2b) experienced a sharp GPP decrease in the model simulations during the monsoon season due to precipitation^31^. The GPP from the model at the cropland site (Fig. S2c) showed the early onset of the spring season due to ample precipitation and model structural uncertainty^30^ (see the “Potential uncertainties and limitations” section). For ecosystem respiration (ER), the cropland site has better performance, with higher R^2^ and IA values of 0.69 and 0.87, respectively (Table 3).

### Ecosystem water-use efficiency (EWUE)

**Figure 1:**
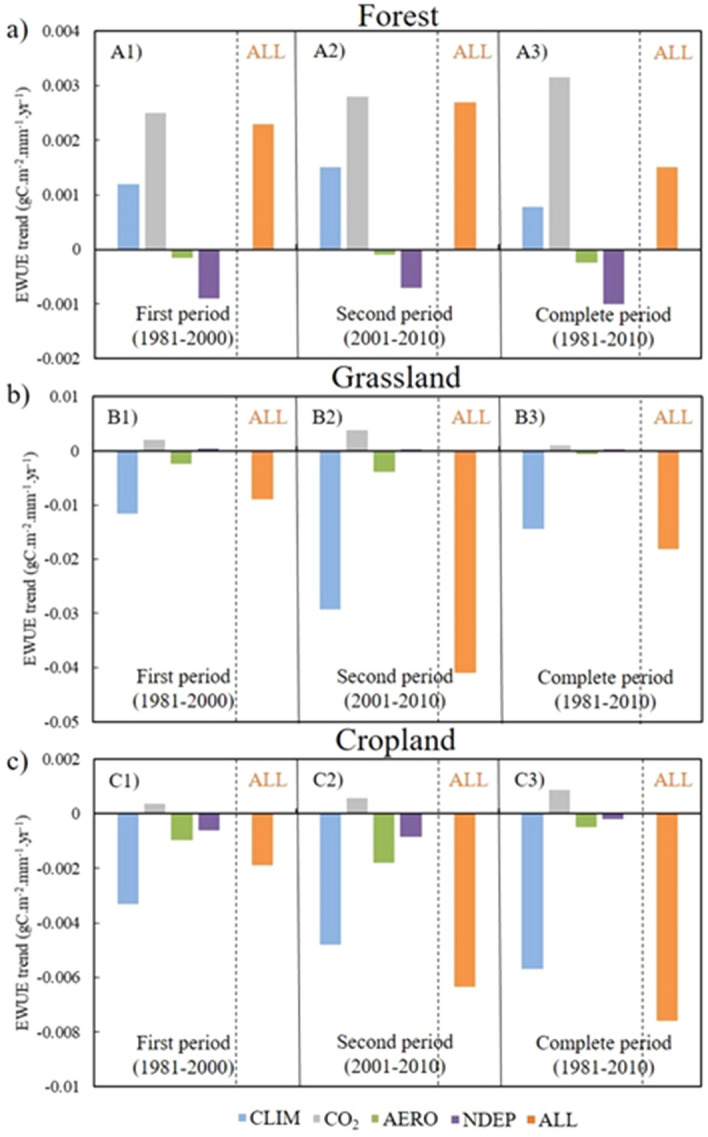
Ecosystem water-use efficiency (EWUE) trends for (**a**) CN-Qia (Evergreen Needleleaf Forest), (**b**) CN-Cng (Grassland), and (**c**) US-Ne3 (Cropland). Blue color represents CLIM (E1) effect, grey represents CO_2_ (E2–E1) effect, green represents aerosol concentration AERO (E3–E1) effect, purple represents nitrogen deposition NDEP (E4–E1) effect, and orange represents combined effect of all environmental factors and climate. A1, B1, and C1 represent first period (1981*–*2000), A2, B2, and C2 represent second period (2001*–*2010), and A3, B3, and C3 represent complete period (1981*–*2010).

Figure 1 shows the EWUE trends (gC m^−2^ mm^−1^ year^−1^) at the three study sites and their variations with climate (E1), CO_2_ (E2–E1), aerosol concentration (E3–E1), and nitrogen deposition (E4–E1) in the growing season. E1 (CLIM) showed different trends for the three sites at multiple periods: in the forest site in 1981–2010 (A3), the value was approximately 0.001 gC m^−2^ mm^−1^ year^−1^ (Fig. 1a), while it decreased in the grassland and cropland sites to − 0.014 and − 0.006 gC m^−2^ mm^−1^ year^−1^, respectively (Fig. 1b, c). This was affected by trends in precipitation, temperature, and solar radiation at each study site (Table 4). In case of CO_2_ (E2–E1), the maximum increasing trend of 0.003 gC m^−2^ mm^−1^ year^−1^ was noted at the CN-Qia (forest) site, with a statistical significance of 99% (*p* < 0.01, Fig. 1a). For the grassland and cropland sites, the trends were 0.001 and 0.0008 gC m^−2^ mm^−1^ year^−1^ respectively, with a statistical significance of 95% (*p* < 0.05, Fig. 1a). The effect of aerosol concentration (E3–E1) showed a significant decreasing trend (*p* < 0.05) at the grassland and cropland sites in the study period (Fig. 1b, c) due to a significant increase in aerosol concentration (Table S4). However, the trend was not significant at the forest site (Fig. 1a). Considering the effect of nitrogen deposition (E4–E1), EWUE showed a significant decreasing trend (*p* < 0.01) at the forest site (due to lack of nitrogen deposition) but the effect was not significant at the other two study sites (Fig. 1). Experiment E5 (ALL, combined effect of climate and environmental factors), showed an increase in EWUE trend at the forest site (0.002, *p* < 0.05) and a decrease at grassland and cropland sites (− 0.018 and − 0.008) both at a statistical significance of 95% (*p* < 0.05, Fig. 1a, Tables S1, S2, and S3).

**Table 4:** Trends for climatic variables such as precipitation, temperature, specific humidity, longwave solar radiations, shortwave solar radiations, wind speed, and surface pressure at three study sites during the growing season (April–August) from 1980 to 2010.

	CN-Qia (Evergreen Needleleaf Forest)		
	1981–2000	2001–2010	1981–2010
Precipitation (mm year^−1^)	4.809	– 3.679*	2.982*
Temperature (K)	0.018	0.012*	0.017*
Specific humidity (g/kg)	0.029	– 0.107	0.039
Longwave solar radiations (W m^−2^)	0.046	– 0.496	0.029
Shortwave solar radiations (W m^−2^)	0.435	0.303*	0.302*
Wind speed (m s^−1^)	0.004	0.004	– 0.002
Surface pressure (Pa)	2.747	4.552	1.246
**CN-Cng (grassland)**			
Precipitation (mm year^−1^)	– 4.484	0.858*	– 4.807*
Temperature (K)	0.038*	0.119*	0.023*
Specific humidity (g/kg)	– 0.047*	– 0.076*	– 0.055
Longwave solar radiations (W m^−2^)	– 0.101	0.149	0.009
Shortwave solar radiations (W m^−2^)	0.463*	1.023*	0.314*
Wind speed (m s^−1^)	– 0.002	– 0.025	– 0.001
Surface pressure (Pa)	– 2.013	3.696	0.272
**US-Ne3 (cropland)**			
Precipitation (mm year^−1^)	– 8.705*	6.693	– 2.338*
Temperature (K)	0.027*	0.173*	0.008*
Specific humidity (g/kg)	– 0.028	0.102	– 0.029
Longwave solar radiations (W m^−2^)	0.033	0.382	0.055
Shortwave solar radiations (W m^−2^)	0.079*	1.268*	0.429*
Wind speed (m s^−1^)	– 0.016	0.017	– 0.014
Surface pressure (Pa)	– 0.685	2.680	0.678

The time periods were from 1981 to 2010 (complete period), 1981 to 2000 (first period), and 2001 to 2010 (second period).

*Denotes results at 0.05 significance level.

### Transpiration water-use efficiency (TWUE)

**Figure 2:**
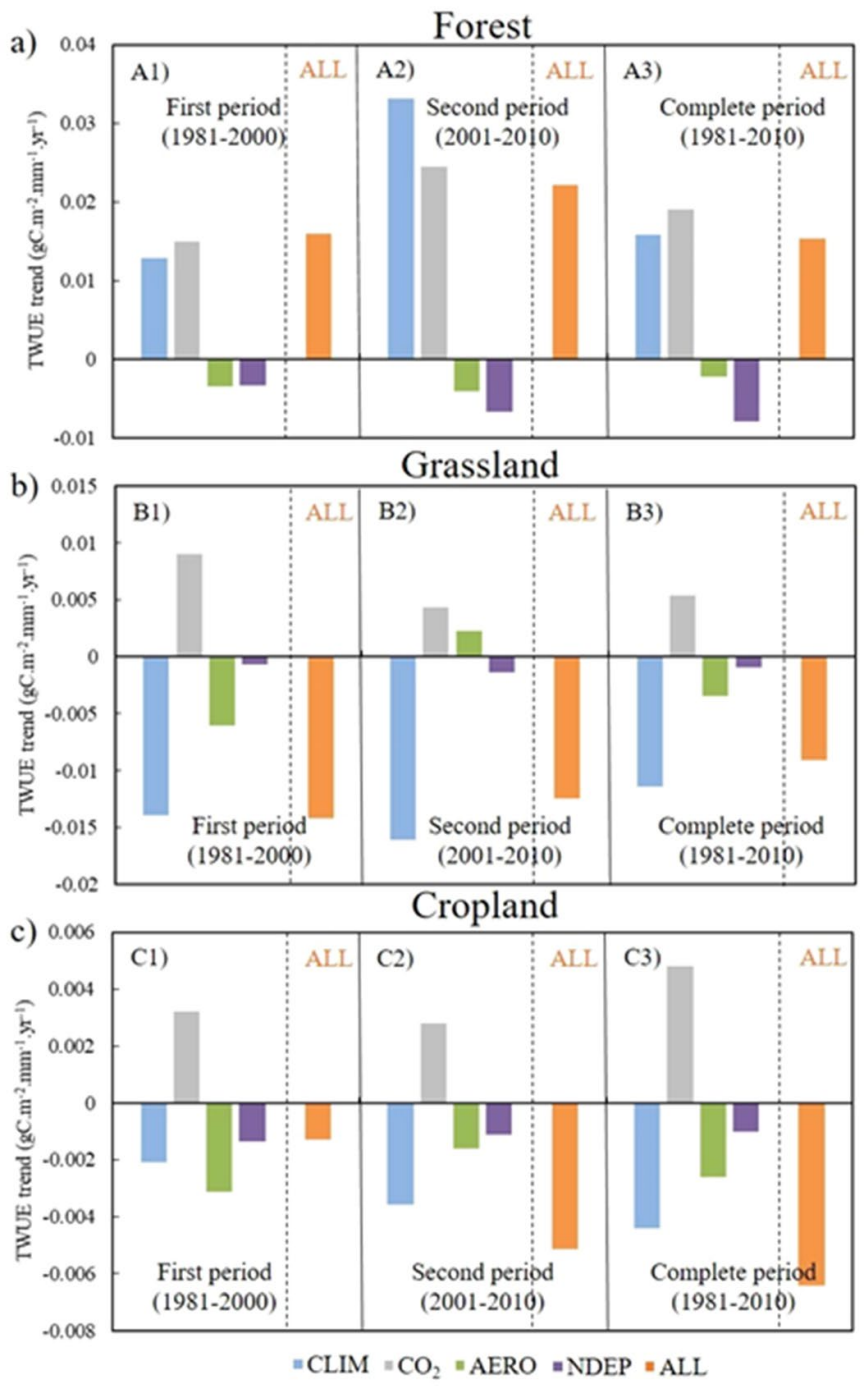
Transpiration based water-use efficiency (TWUE) trends for (**a**) CN-Qia (Evergreen Needleleaf Forest), (**b**) CN-Cng (Grassland), and (**c**) US-Ne3 (Cropland). Blue color represents CLIM (E1) effect, grey represents CO_2_ (E2–E1) effect, green represents aerosol concentration AERO (E3-E1) effect, purple represents nitrogen deposition NDEP (E4–E1) effect, and orange represents combined effect of all environmental factors and climate. A1, B1, and C1 represent first period (1981*–*2000), A2, B2, and C2 represent second period (2001*–*2010), and A3, B3, and C3 represent complete period (1981*–*2010).

Figure 2 shows the TWUE for the three study sites. In the case of E1 (CLIM) in Fig. 2b, c, TWUE followed the same increasing trend (0.016 gC m^−2^ mm^−1^ year^−1^, Fig. 2a) as EWUE for the forest site but decreased for the grassland and cropland sites (− 0.011 and − 0.004 gC m^−2^ mm^−1^ year^−1^, respectively). The significant increasing trend for the CO_2_ (E2–E1) effect was observed for the forest and cropland sites (− 0.019 and − 0.003 gC m^−2^ mm^−1^ year^−1^, respectively); however, the grassland site showed non-significant trend. The aerosol concentration significantly increased at the grassland and cropland sites in the study periods, which decreased the TWUE trends (E3–E1) significantly for both sites (− 0.003 gC m^−2^ mm^−1^ year^−1^ for both, Tables S2, S3, and S4). The decreasing trend for NDEP was significant at the forest site (− 0.002, Table S4) and ultimately induced the significant decreasing trend for TWUE (E4–E1, − 0.008 gC m^−2^ mm^−1^ year^−1^). However, the effect of NDEP was not significant at the grassland and cropland sites similar to EWUE. The TWUE for the effect of combined factors (E5) showed significant increasing trends at the forest site (0.015 gC m^−2^ mm^−1^ year^−1^). However, significant decreasing trends were observed at the grassland and cropland sites over the complete study period (− 0.009 and − 0.006 gC m^−2^ mm^−1^ year^−1^, respectively).

### Inherent water-use efficiency (IWUE)

**Figure 3:**
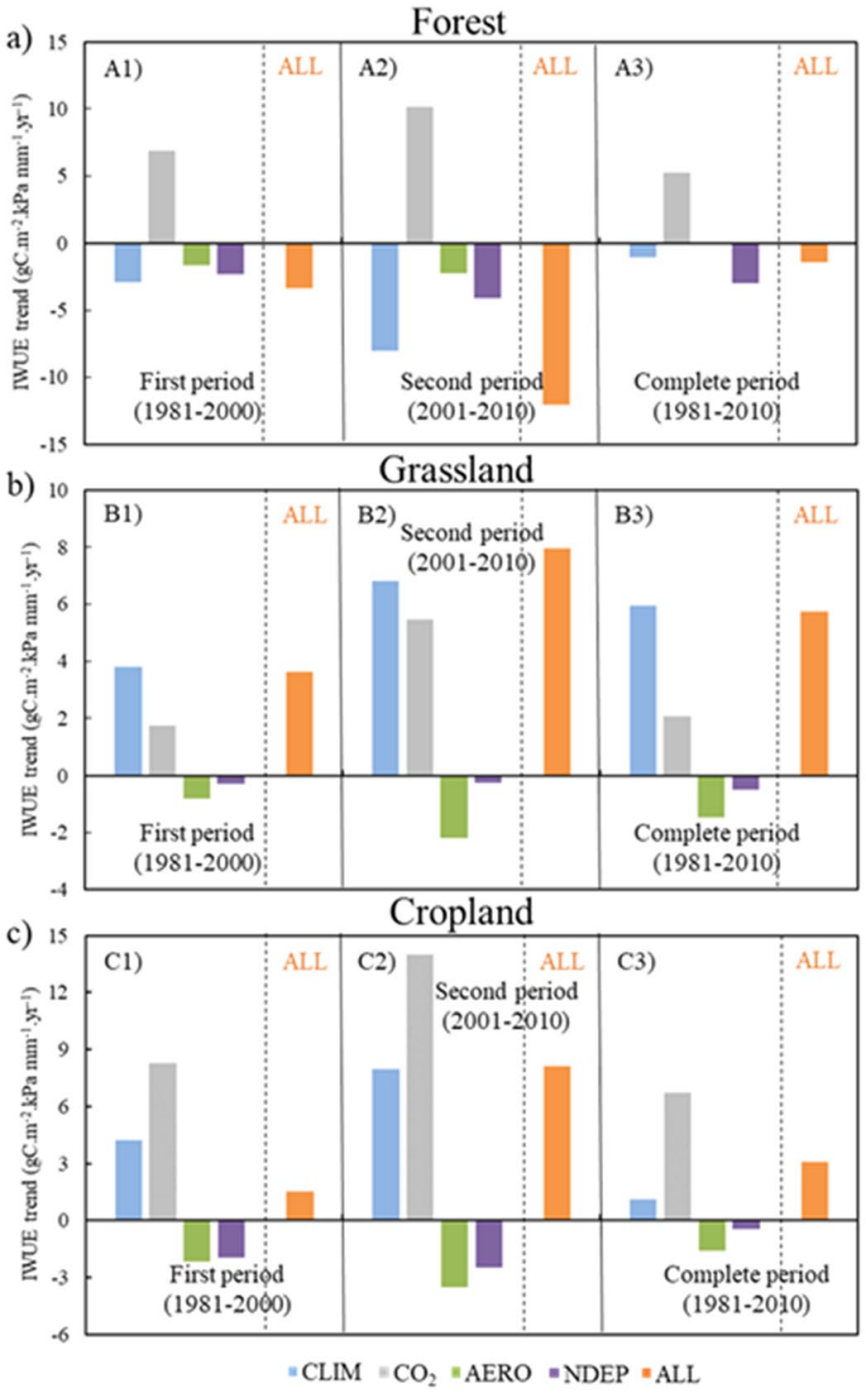
Inherent water-use efficiency (IWUE) trends for (**a**) CN-Qia (Evergreen Needleleaf Forest), (**b**) CN-Cng (Grassland), and (**c**) US-Ne3 (Cropland). Blue color represents CLIM (E1) effect, grey represents CO_2_ (E2–E1) effect, green represents aerosol concentration AERO (E3–E1) effect, purple represents nitrogen deposition NDEP (E4–E1) effect, and orange represents combined effect of all environmental factors and climate. A1, B1, and C1 represent first period (1981*–*2000), A2, B2, and C2 represent second period (2001*–*2010), and A3, B3, and C3 represent complete period (1981*–*2010).

The IWUE showed higher trends than EWUE and TWUE due to the inclusion of VPD (Fig. 3). A significant decreasing IWUE (E1) was observed at the forest site (− 1.077 gC m^−2^ kPa mm^−1^ year^−1^), while a significant increase was seen at the grassland and cropland sites (5.934 and 1.089 gC m^−2^ kPa mm^−1^ year^−1^, respectively). An increasing trend for IWUE was observed for the effect of CO_2_ concentration (E2-E1) at all three (forest, grassland, and cropland) sites (5.228, 2.058, and 6.726 gC m^−2^ kPa mm^−1^ year^−1^, respectively). The trend for IWUE due to effect of aerosol concentration (E3–E1) showed a similar pattern to those for EWUE and TWUE, and was non-significant at the forest sites and significant decreasing trend at the grassland and cropland sites (− 1.463 and − 1.5876 gC m^−2^ kPa mm^−1^ year^−1^, respectively). For the effect of nitrogen deposition (E4–E1), a significant decreasing trend was observed for the forest sites (− 2.979 gC m^−2^ kPa mm^−1^ year^−1^), while, the grassland and cropland sites showed non-significant trends. The IWUE for the E5 experiment (all factors combined), showed significant decrease at the forest site (− 1.467 gC m^−2^ kPa mm^−1^ year^−1^) and significant increasing trend at the grassland and cropland sites (5.735 and 3.095 gC m^−2^ kPa mm^−1^ year^−1^), opposite to those of EWUE and TWUE (Tables S1, S2, and S3).

## Discussion

### Effects of multiple environmental factors on WUE

An increasing EWUE trend was observed at the forest site with a change in climate (E1) which might be due to the increasing trend in precipitation (2.982 mm year^−1^) that enhanced carbon assimilation over the 30 years (Table 4). However, as precipitation decreased at the grassland and cropland sites (− 4.807 and − 2.338 mm year^−1^), it reduced carbon assimilation as well (Table 4), which in turn negatively affected EWUE. This relationship between precipitation and carbon assimilation is supported by the results of a previous study by Sun et al.^30^ and Zhao et al.^32^. The EWUE trend (E1) at the forest site in the first period (1981–2000, A1 in Fig. 1a) was lower than in the second period (2001–2010, A2 in Fig. 1a), indicating an increase in GPP due to rising temperature. The cropland and grassland sites (Fig. 1b, c) showed an increasingly negative trend in EWUE (in contrast to the forest site), as they were affected by climatic variables such as precipitation, temperature, and solar radiation (Table 4).

Due to rising CO_2_ (E2–E1), EWUE increased at all three sites in both study periods due to its structural effect (where plant growth increased due to higher CO_2_ concentration and changed the plant structure with increasing LAI)^3,31^ (Fig. 1). The EWUE trends decreased due to the effect of aerosol concentration (E3-E1) at grassland and cropland sites. ET and carbon assimilation decreased due to the higher concentration of aerosols, which reduced the solar radiation reaching the earth's surface^23,31^ (Table S4). The effect of nitrogen deposition at the forest site decreased in the second period (2001–2010, A2 in Fig. 1a, Table S4) but continued to exhibit a negative trend.

Experiment E5 (ALL), which considered all climate and environmental factors, showed an increase in EWUE at the forest site, which was highly affected by CO_2_ fertilization and increased precipitation over the 3 decades (Fig. 1a, Table 4). However, at the grassland and cropland sites, EWUE decreased due to the negative precipitation trend and the positive trends in temperature and shortwave radiation over the 30-year period (Fig. 1b,c; Table 4). EWUE increased between the two study periods at the forest site but decreased at the grassland and cropland sites, likely due to the increase in CO_2_ and the effects of climatic variables.

In Fig. 2, the trends for TWUE (Case E1, ‘CLIM’) were steeper than those of EWUE, due to lower T_r_ than ET and higher GPP in the forest ecosystem (Figs. 2a, S3). However, the negative trend of TWUE was lessened due to the minimal T_r_ effect in ET at the grassland site, corroborated by ET and T_r_ trends (Figs. 2b, S4). The cropland site also showed a slight reduction in the negative trend of TWUE compared with EWUE due to higher positive trend in T_r_ compared with ET (Figs. 1c, 2c, S5). The CO_2_ fertilization effect (E2–E1) played an important role in the increase in carbon assimilation at the forest site and ultimately increased the TWUE trend compared with those at the grassland and cropland sites with statistical significance (*p* < 0.01) due to a higher LAI at the forest site^2^ (Fig. 2).

Aerosol concentration (E3–E1) and nitrogen deposition (E4–E1) had little effect on the variability of TWUE at all three sites compared with climate (E1) and CO_2_ concentration (E2-E1) (Fig. 2). The effect of aerosol concentration on TWUE was similar to that on EWUE at all three sites, as described previously. As for the effect of nitrogen deposition (E4-E1), the forest site showed a significant decrease (*p* < 0.01) in TWUE due to lack of nitrogen deposition for overall 3 decades (Fig. 2a, Table S4), but no significant trend was observed at the other two sites. The limiting effect of lack of nitrogen deposition on carbon assimilation corroborated the findings of previous studies^15, 33^.

Combining the effects of all variables on TWUE (E5), the trend increased at the forest site and decreased in the grassland and cropland, the same pattern as for the EWUE; however, the magnitude varied across sites due to the effect of T_r_ (Fig. 2). The first (1981–2000) and second (2001–2010) time periods exhibited the same trends for TWUE as for EWUE (increasing at forest and decreasing at cropland) except at the grassland site. It caused by abnormal behavior of aerosol concentration at the grassland site [which showed a decreasing trend in EWUE (Fig. 1b, E3–E1) and an increasing trend in TWUE (Fig. 2b, E3–E1)] and reduces the negative trend in TWUE for all factors (E5) in the second period compared with the first (Fig. 2b).

Figure 3 shows the IWUE at the three study sites. The effects of climate ‘CLIM’ showed opposite trends with respect to EWUE and TWUE. VPD, which is a function of air temperature, has a strong relationship with WUE. Here, the effects of VPD were removed from the analysis by introducing it only in IWUE^15,26^. This phenomenon explains the climate warming pattern especially at the forest and cropland sites, which directly affected plant growth by increasing VPD. The effect of CO_2_ fertilization (E2–E1) positively affected the IWUE due to an increase in atmospheric CO_2_ concentration.

In IWUE, the effects of aerosol and nitrogen deposition followed similar patterns to those of EWUE and TWUE (Fig. 3). The overall trend (E5) showed a decrease in IWUE at the forest site and increases in grassland and cropland sites due to the effects of climatic variables (Table 4). For both study periods, the trends in IWUE (E1 and E5) remained same at all three study sites but changed signs from positive to negative and vice versa compared with EWUE and TWUE due to removal of the VPD effect (Fig. 3).

### Impact of climatic variables on WUE and its implications

Table 5 shows the partial correlations between multiple WUE terms and climatic variables (precipitation, solar radiation, and temperature). For the forest site (CN-Qia), EWUE and TWUE were positively correlated with all three climatic variables. Especially, the shortwave solar radiation showed a significant partial correlation with EWUE and TWUE (0.532 and 0.579, respectively, *p* < 0.05; Table 5). Solar radiation increased photosynthesis and carbon assimilation, but a higher LAI reduced the amount of radiation reaching the surface and ultimately reduced soil evaporation at the forest site^15^. Increased climate warming created drought-like conditions with decreasing precipitation trends (– 3.679 mm year^−1^) over the last 10 years of the study period at forest site (2001–2010, Table 4). However, deeper roots of the forest trees could withdraw water from greater depths and were less affected by the lack of precipitation^10^; this resulted in a positive EWUE trend (Fig. 1).

**Table 5:** Partial correlation between water-use efficiency (WUE) terms and climatic variables (precipitation, shortwave solar radiations, and temperature).

		Precipitation	Solar radiations (shortwave)	Temperature
CN-Qia (evergreen needleleaf forest)	EWUE	0.007	0.532**	0.016
	TWUE	0.223	0.579**	0.104
	IWUE	– 0.085	– 0.516**	– 0.299
CN-Cng (grassland)	EWUE	0.401**	0.035	– 0.499**
	TWUE	0.264	0.146	– 0.414*
	IWUE	– 0.066	– 0.163	0.003
US-Ne3 (cropland)	EWUE	0.111	– 0.451*	-0.135
	TWUE	0.328*	– 0.826**	– 0.276
	IWUE	0.012	0.778**	0.268

EWUE, TWUE, IT-WUE = Ecosystem, Transpiration, and Inherent WUE.

**0.05 significance level; *0.1 significance level.

At the grassland site, EWUE was somewhat correlated with precipitation (0.4010, *p* < 0.05; Table 5) but not with solar radiation and showed a significant negative correlation with temperature (– 0.499, *p* < 0.05; Table 5). Precipitation at the grassland site showed a decreasing trend (– 4.807 mm year^−1^ for 1981–2010, Table 4) with relatively low mean annual temperature (~ 4.9 °C) and precipitation (400 mm, Table 1). These conditions represented a water stress environment with low LAI (due to less precipitation), which also reduced carbon assimilation. However, the last 10 years showed a little increase in the precipitation trend for grassland (Table 4), which did not affect the decreasing trend in carbon assimilation and ET and further decreased the negative EWUE trend possibly due to dry conditions (Fig. 1b).

For the cropland site, a fairly significant negative partial correlation was observed for EWUE with solar radiation (– 0.451, *p* < 0.1; Table 5). An increase in solar radiation caused higher ET (due to no water stress) and lower EWUE. This phenomenon was stronger for TWUE, which had a much higher negative partial correlation (– 0.826, *p* < 0.05; Table 5). A negative partial correlation was also observed with temperature, as rising temperature (0.008 K) increased ET at the cropland site which decreased EWUE and TWUE trends (Table 5, Figs. 1c, 2c). The decreasing trend of precipitation (– 2.338 mm year^−1^; Table 4) had little effect on EWUE and showed a non-significant partial correlation value (0.111), likely due to no water stress at the cropland site (Table 5).

For all three sites, IWUE showed opposite trends (positive to negative in case of forest site and negative to positive in case of grassland and cropland sites) to EWUE and TWUE because the effect of VPD (which is a function of temperature) was removed (Fig. 3). The results reflect a warming pattern at forest and cropland sites, and the grassland site showed an increasingly drier climate. Other climatic variables, such as specific humidity, longwave solar radiations, wind speed, and surface pressure, did not show significant trends at any site (Table 4).

### Implications of WUE over multiple LCTs

WUE is the ratio between the water used in plant metabolism (or the amount of carbon uptake) to water loss from plants^34^. It is considered to be an important index for the study of the increase in atmospheric CO_2_ concentration, its effects on the ecosystem, and the changing climate with significant warming conditions^35^. Due to the increase in irrigation activities, the land use land cover changes have disturbed the carbon and water cycle. These disturbances to the terrestrial ecosystem increased the EWUE at the forest ecosystem and exhibited the greening of the land surface. Another reason might be an increase in atmospheric CO_2_ available to the plants which increases the rate of GPP^2^ (Fig. S3). However, in grassland and cropland conditions, the EWUE decreases that might have serious concerns for the warming conditions (Figs. S4, S5). In the forest ecosystem, the rate of water gain (carbon uptake) was higher than the rate of water loss by plants. However, the opposite trends were observed at the grassland and cropland sites (Figs. S4, S5). When the rate of water loss from plants was higher, the ecosystem moved to drier conditions, causing droughts in the long run. The increasing CO_2_ level also caused warmer conditions, which increased ET and led to water stress for plants.

The three LCTs in this study showed different trends in WUE under similar increasing trends for temperature and shortwave solar radiation over the last 3 decades. The different climate zones and soil characteristics associated with each site (Cfa-warm temperate for forest site, Bsk-arid for grassland, and Dfa-boreal for cropland, Table 1) affect their WUE trends. The forest and cropland sites showed a shifting of climate to warmer conditions (Table 4, Figs. S3 and S5); however, the grassland site showed a drier climatic pattern with significant decreasing precipitation trend and the increasing trend for temperature and solar radiations. Considering the effect of albedo, forests have a lower albedo than grassland and cropland ecosystems, which induced a summer cooling effect^10,36^. However, this (cooling effect) occurred due to the carbon loss to the atmosphere and reduction in carbon sink to terrestrial ecosystems from the atmosphere, which is reflected in increasing EWUE (E5) and TWUE (E5) trends at the forest site and decreasing trends at cropland and grassland sites^37^ (Figs. 1, 2). The anthropogenic increase of CO_2_ in the atmosphere, more than any other factor caused an increase in EWUE and TWUE (Figs. 1, 2), moreover, the reduction in carbon sink due to land cover changes leaves more carbon in the atmosphere and induced further warming^38^. The increased temperature due to water stress can lead to a reduction in soil moisture and ET, which can increase vegetation mortality^13,39,40^.

Forest and cropland sites respond differently to water stress. In drought conditions, forests can have green canopies for longer periods than croplands because trees can access deeper stored soil water; this ultimately reduces the effects of drought and high temperature over forests^10^. Here, the two study periods (1981–2000 and 2001–2010) showed increasing significant trends for temperature and shortwave solar radiations at all three sites (Table 4). At the forest site, the second period (2001–2010) showed higher trends for ‘E5’ in EWUE and TWUE than the first period (1981–2000) due to elevated CO_2_ in the atmosphere, which increased carbon assimilation with the increase in temperature (Figs. 1a, 2a, S3; Table 4). The grassland site showed a decrease in EWUE (E5) in the second period (2001–2010) compared with the first (1981–2000) due to negative trends of climate effect and aerosol concentration (Figs. 1b, 2b) as explained in the previous section. The cropland site (Figs. 1c, 2c) showed a decrease in EWUE (E5) and TWUE (E5) trends in the second period (2001–2010) compared with the first period (1981–2000) because of lower carbon assimilation and higher ET caused by the rise in temperature (0.027 K in the first period and 0.173 K in the second period; Table 4). EWUE and TWUE at all sites (Figs. 1, 2) showed their maximum trends (increasing/decreasing for forest/grassland and cropland, respectively) for E5 during the 2001–2010 period due to increased warming induced by rising CO_2_ in the atmosphere and other climatic variables^38^.

Due to increasing temperature, VPD and demand for ET both increased^29^. This phenomenon is also supported by Fig. 3, in which the IWUE exhibits opposite trends (compared with EWUE and TWUE in E1 and E5 experiments; positive to negative trends in case of forest site and negative to positive trends in case of grassland and cropland sites) for all sites when the effect of VPD was removed. These results have implications for climate due to rising CO_2_ and different feedback mechanisms to the atmosphere from various LCTs.

### Potential uncertainties and limitations

Quantification of uncertainty in earth system models for simulation of ecological processes plays a critical role in the authentication of results^41^. The sources of uncertainty in LSMs include model uncertainty, climate data (forcing) uncertainty, and initial conditions uncertainty^42,43^. The LSM used in this study was the CLM5.0, which was updated regularly on the basis of model parameterization and uncertainties previously defined by scientists; however, multiple processes involved in the carbon and water cycles can generate additional uncertainties^44–47^. Figure S2c shows how a model estimation of GPP over cropland could be affected by an early onset of the spring season (model structural uncertainty). The grid-scale forcing data GSWP3v1 were used in this study, which also introduced uncertainties in the estimation of carbon and water cycle fluxes. This was corroborated by previous literature^48,49^. Figure S2b shows that the GPP estimation from the model was sensitive to precipitation (forcing data uncertainty). The carbon cycle requires a large number of spin-up cycles to reach equilibrium, and this affected the initial values of the parameters, which added to the overall uncertainty of the model^43^. In land carbon uptake, model structural uncertainty is predominant among all possible uncertainties^41^. Lovenduski and Bonan^50^ explained that there is a limit below which the uncertainty cannot be further reduced.

Limitations are common in modeling studies. Initially, the coarse resolution of forcing data (climatic variables) from GSWP3v1 was 0.5°. High resolution data or weather station/flux tower data are more reliable for site level studies. Due to non-availability of long-term site level data (1981–2010), gridded 0.5° GSWP3v1 data were used in the study. Secondly, very few studies have used the bio-geochemical cycle of CLM5.0, which needs to be analyzed further to enhance the accuracy of the model, especially at cropland regions. The site scale of the study was also one of the limitations, as the regional or global scale could better analyze the effects of multiple land cover types on the climate due to rising CO_2_ and the influence of nitrogen deposition and aerosol concentration. Future studies should be conducted at a regional to global scale to study this phenomenon in detail over multiple regions.

## Conclusions

In this study, carbon and water fluxes at three sites with different LCTs such as forest, grassland, and cropland were estimated using CLM5.0, a state-of-the-art LSM. The effects of climate and other environmental factors such as rising atmospheric CO_2_, aerosol concentration, and nitrogen deposition were evaluated using three WUE definitions (EWUE, TWUE, and IWUE). Multiple definitions of WUE over the 30-year time period with the effect of multiple LCTs and their feedbacks to the changing climate were discussed. The model was validated with flux tower data. The best results for ET were obtained at the cropland site, with the highest R^2^ value of 0.74; however, the forest site showed the best results in terms of GPP estimation, with lower RMSE and bias values (2.08 and – 0.04 gC m^−2^ day^−1^, respectively) and R^2^ value of 0.71.

The increasing trends in EWUE and TWUE at the forest site reflected a strong CO_2_ fertilization effect, which continued through the two study periods (1981–2000 and 2001–2010). However, the decreasing trends in EWUE and TWUE at the grassland and cropland sites over the same two periods represented a decrease in GPP and an increase (decrease) in ET at cropland (grassland) due to the rise in temperature and CO_2_-induced climate warming. This resulted in water stress at the grassland site, which was supported by the trends in climatic variables (precipitation, temperature, and shortwave solar radiations), ET, and GPP and altered the water and carbon cycles. These trends in EWUE and TWUE at multiple LCTs have strong implications for climate warming at the forest and cropland sites, while the grassland site showed a drier climate with a low impact of climate warming. The opposite IWUE trends (opposite to EWUE and TWUE at all sites) confirmed that the carbon and water fluxes were controlled by the rise in temperature (with the inclusion of VPD in IWUE).

The rise in CO_2_, temperature, and water availability were the three most important factors affecting WUE at all LCTs. Lack of nitrogen deposition reduced carbon assimilation, especially at the forest site, and the first decade of the twenty-first century (2001–2010) showed maximal trends in multiple WUE definitions due to increase in temperature and climate effect induced by increasing atmospheric CO_2_ at all sites. These results show that future climate warming can result from the anthropogenic increase in atmospheric CO_2_ concentration at forest and cropland sites. Therefore, further works are required at the global and continental scales to evaluate the impacts of climate warming and other environmental factors at multiple LCTs to devise mitigation plans essential in the policy-making domain.

## Data and methods

### Study area and datasets

This study was conducted at three flux tower sites, CN-Qia, CN-Cng, and US-Ne3, located in Ji’an, China, Changling, China, and Nebraska, USA, respectively, to evaluate the effects of water and carbon fluxes caused by climate and environmental factors (Table 1). The sites were selected based on LCT (forest, grassland, and cropland) to assess the impacts on climate, and vice versa, using a modeling approach. Only sites with at least 3 years of data and less than 10% missing data were selected for validation purposes. Energy balance closure was also calculated, and all sites showed less than 10% closure, which is acceptable as corroborated by previous studies^51, 52^.

Data from the three flux tower sites were obtained from the official website of the FLUXNET network^53^, and the meteorological details are given in Table 1. The water and carbon flux variables used were GPP, ER, ET, and precipitation.

### Model description and experimental design

We used CLM5.0 to evaluate the water and carbon fluxes. This is the most recent version of CLM, released by the National Center for Atmospheric Research (NCAR) in May 2018. The new model used updated hydrology parameterization with inclusion of dry surface layer-based soil evaporation resistance and revised canopy interception parameterization^54^. For stomatal conductance, we used the Medlyn conductance model^55^ for its realistic behavioral low humidity levels, instead of the Ball–Berry model used in the previous CLM version. Moreover, potential photosynthesis was replaced with nitrogen-limited photosynthesis^56^.

The default data with CLM5.0 for nitrogen deposition and aerosol concentration were used in the model. The data were created using the Whole Atmosphere Community Climate Model^57^ (WACCM) with the NCAR Community Earth System Model (CESM) under the Coupled Model Inter-comparison Project (CMIP6) historical simulations from 1849 to 2015 on a monthly timescale at a global level. The site level data were extracted by CLM5 tools and scripts. Table S4 shows the variation trends of aerosol concentration and nitrogen deposition.

The model was analyzed from 1951 to 2010 using GSWP3v1 forcing data that included seven meteorological variables of precipitation, solar radiation (shortwave, longwave), air temperature, humidity, wind speed, and atmospheric pressure. A total of 12 runs of 60-year simulations were conducted (720 years); the first 11 runs were used to spin-up the model for parameters to reach the equilibrium stage, and the last run was used for analysis. From the last run, 1981–2010 was selected due to the occurrence of extreme weather events in those last 3 decades^58,59^.

We conducted five experiments at each site.

*E1 (CLIM)* Transient climate with all other factors constant. In this experiment, the effect of climate change from 1981–2010 was observed by evaluating GPP, ET, and T_r_ using WUE. The other factors CO_2_, aerosol concentration, and nitrogen deposition were fixed at 1981 values, leaving only climate to vary over the 3 decades (Table 2).

*E2 (CLIM + CO*_*2*_*)* Transient climate and CO_2_ concentration while all other factors constant. With varying climate, the increase in CO_2_ was also considered in this experiment, while the remaining two factors including aerosol concentration and nitrogen deposition were fixed at their 1981 values. The individual effect of CO_2_ increase on WUE were evaluated by subtracting the E1 experiment results from E2 (Table 2).

*E3 (CLIM + AERO)* Transient climate and aerosol concentration and all other factors constant. Climate variations including aerosol concentration values from the 1981 to 2010 was evaluated by using WUE, while CO_2_ and nitrogen deposition values were constant at 1981 levels. The individual effect of aerosol concentration on WUE was evaluated by subtracting E1 results from E3 (Table 2).

*E4 (CLIM + NDEP)* Transient climate and nitrogen deposition and all other factors constant. This experiment evaluated the effect of nitrogen deposition along with varying climate by using WUE, while CO_2_ and aerosol concentration was constant at their 1981 values. The individual effect of nitrogen deposition was evaluated by subtracting E1 results from E4 (Table 2).

*E5 (CLIM + CO*_*2*_* + AERO + NDEP)* All transient factors. This last experiment was conducted to examine the combined effect of change in climate and all environmental factors (CO_2_, aerosol concentration, and nitrogen deposition) on WUE in the last 3 decades (1981–2010; Table 2).

The five experiments were performed at all the three sites individually to analyze the effects of climate change and environmental factors over a 30-year period (1981–2010) at forest, grassland, and cropland sites.

### Analysis

Three definitions of WUE, characterized by ET, T_r_, and VPD, were used in the study for differentiating the effects on the ecosystems with multiple LCTs (forest, grassland, and cropland). The study period (1981–2010) was divided into two time periods, 1981–2000 and 2001–2010, to further classify the effects of climate and other environmental factors on the ecosystems in a continuously changing environment. EWUE, TWUE, and IWUE were corroborated from previous studies^8,15^, as follows:

1 $$EWUE = GPP/ET$$

2 $$TWUE = GPP/T_{r}$$

3 $$IWUE = (GPP/T_{r} ) \times VPD$$

where *GPP* refers to annual gross primary production (gC m^−2^), ET to annual evapotranspiration (mm), *T*_r_ to annual transpiration (mm), and *VPD* to vapor pressure deficit (kPa) of the growing season from April to August (5 months).

Nonparametric Mann–Kendall tests were used to calculate the monotonic trends in all the experimental results from the model^60,61^. The null hypothesis (H_0_) assumed that the data were randomly ordered, independent, and showed no significant trends and was rejected if the *p*-value of the test statistic was less than the 0.05 significance level. The trend of each data series (slope) was estimated based on a formulation developed by Sen^62^, wherein the median of the slopes was estimated based on each pair in the data series. To compare the variability of multiple WUE values terms in response to climatic variables (precipitation, temperature, and solar radiation), partial correlation analyses were performed for all three study sites. They provided the correlation between each of the three WUE terms and the three climatic variables while controlling the other two.

Other error matrices, including the R^2^, RMSE, slope, bias, and IA, were calculated to validate the model results with the flux tower data^63,64^. Slope is calculated using the linear regression equation. Calculations of the error matrices were carried out using the following formulas:

4 $$R^{2} = \frac{{\left( {n\left( {\sum\limits_{i = 1}^{n} {O_{i} C_{i} } } \right) - \left( {\sum\limits_{i = 1}^{n} {O_{i} } } \right)\left( {\sum\limits_{i = 1}^{n} {C_{i} } } \right)} \right)^{2} }}{{\left[ {n\sum\limits_{i = 1}^{n} {O_{i}^{2} } - \left( {\sum\limits_{i = 1}^{n} {O_{i} } } \right)^{2} } \right]\left[ {n\sum\limits_{i = 1}^{n} {C_{i}^{2} } - \left( {\sum\limits_{i = 1}^{n} {C_{i} } } \right)^{2} } \right]}}$$

5 $$RMSE = \sqrt {\sum\limits_{i = 1}^{n} {(C_{i} - O_{i} )^{2} /n} }$$

6 $$Bias = \sum\limits_{i = 1}^{n} {(C_{i} - O_{i} )/n}$$

7 $$IA = 1 - \frac{{\sum\limits_{i = 1}^{n} {(C_{i} - O_{i} )^{2} } }}{{\sum\limits_{i = 1}^{n} {(|C_{i} - \overline{O} | + |O_{i} - \overline{O} |)^{2} } }}$$

where $C_{i}$ and $O_{i}$ represent the calculated and observed values respectively, and $\overline{O}$ denotes the mean of the observed data.

## Supplementary information

Supplementary Information.
