# Supplementary material for: Impact of climate, rising atmospheric carbon dioxide, and other environmental factors on water-use efficiency at multiple land cover types
Source: Sci Rep. 2020 Jul 15;10:11644. doi: 10.1038/s41598-020-68472-7 (PMC7363916; doi:10.1038/s41598-020-68472-7)
Supplement: Supplementary file 1 — Supplementary Information. [file 41598_2020_68472_MOESM1_ESM.docx]

**Impact of Climate, Rising Atmospheric Carbon Dioxide, and other Environmental Factors on Water-Use Efficiency at Multiple Land Cover Types**

**Muhammad Umair^1^, Daeun Kim^2^, and Minha Choi^1,*^**

*^1^* Environment and Remote Sensing Laboratory, Dept. of Water Resources, Graduate School of Water Resources, Sungkyunkwan University, Suwon 16419, Republic of Korea

*^2^* Dept. of Civil & Environmental Engineering, Sungkyunkwan University, Suwon 16419, Republic of Korea

[mhchoi@skku.edu](mailto:mhchoi@skku.edu)


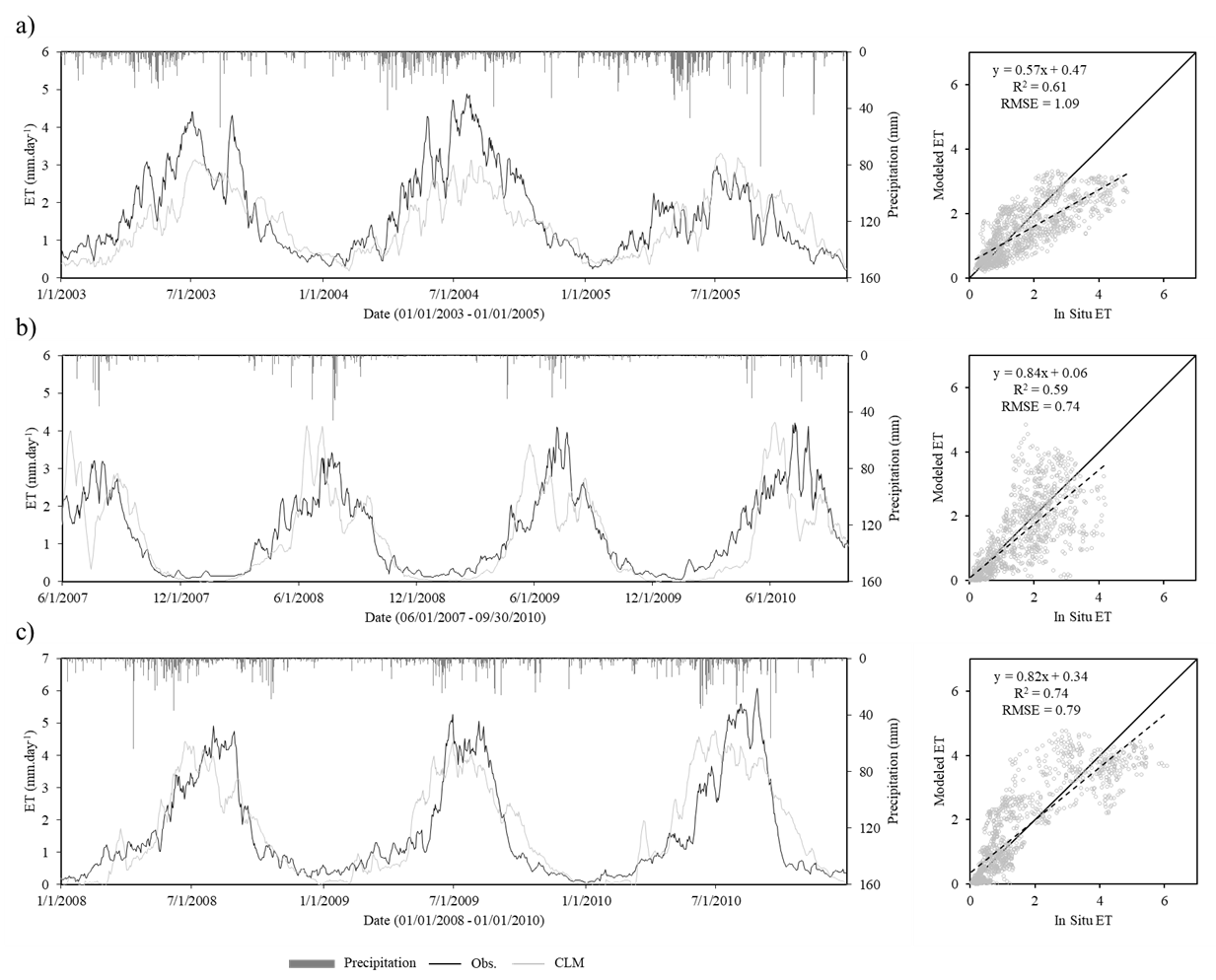


**Figure S1.** Time series and scatter plots of ET from CLM 5.0 with FLUXNET observations at a) CN-Qia (Evergreen needleleaf forest), b) CN-Cng (Grassland), and c) US-Ne3 (Crops).


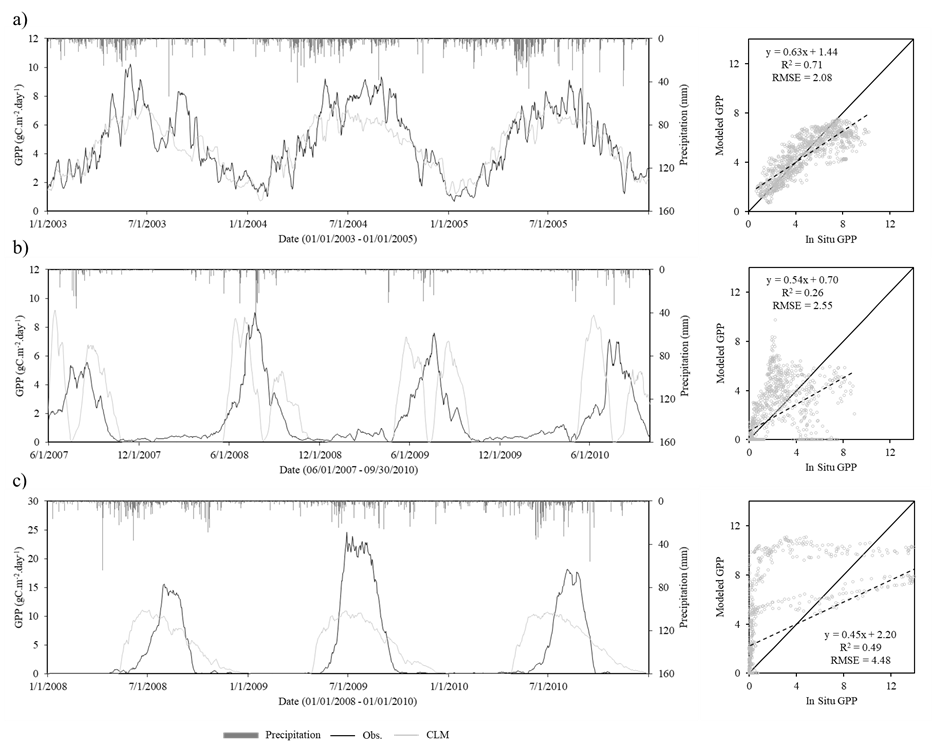


**Figure S2.** Time series and scatter plots of GPP from CLM 5.0 with FLUXNET observations at a) CN-Qia (Evergreen needleleaf forest), b) CN-Cng (Grassland), and c) US-Ne3 (Crop)


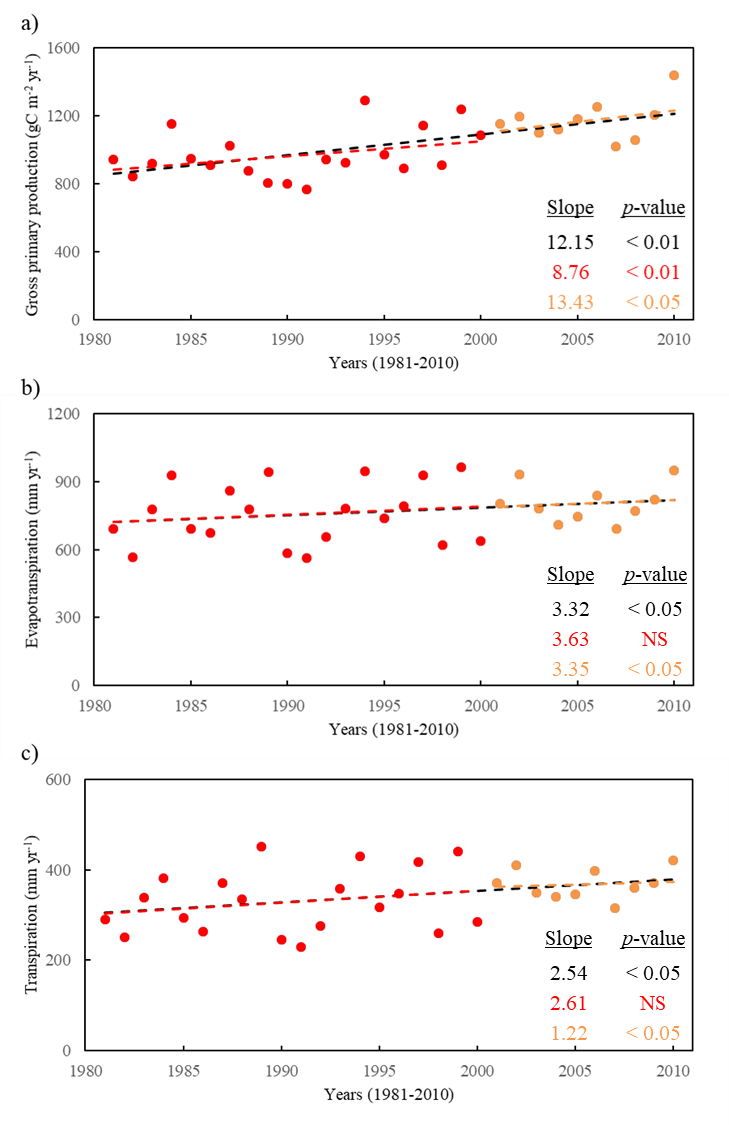


**Figure S3.** Time series with trend lines, slope, and *p*-values for the three components (GPP, ET, and T_r_) of WUE at the CN-Qia site. Red data are from the first time period (1981–2000), orange data are from the second time period (2001–2010), and black represents the complete time period (1981–2010).


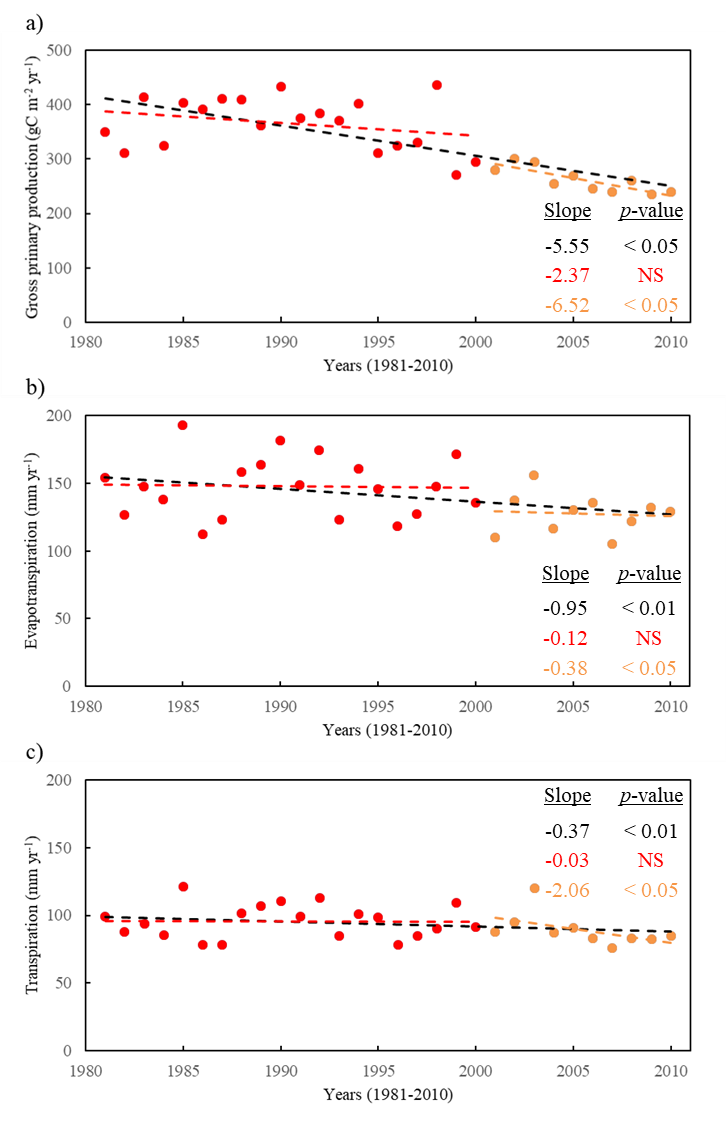


**Figure S4.** Time series with trend lines, slope, and *p*-values for the three components (GPP, ET, and T_r_) of WUE at the CN-Cng site. The red color refers to the first time period (1981–2000), orange refers to the second time period (2001–2010), and the black represents the complete time period (1981–2010).


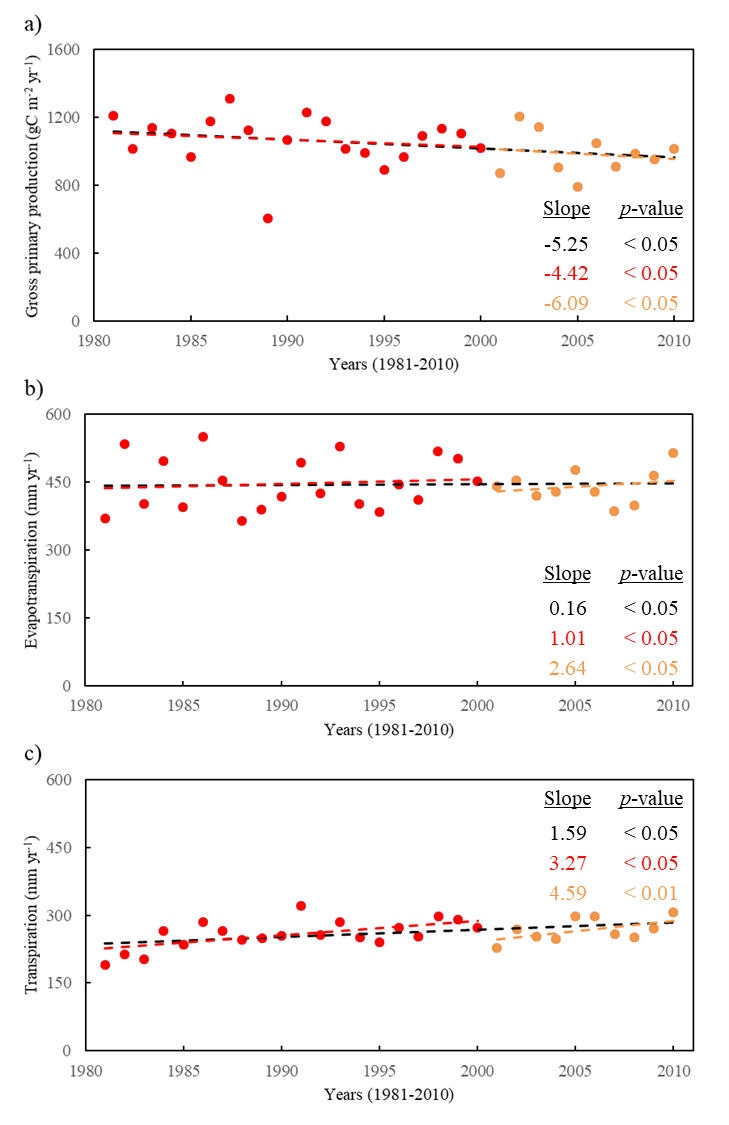


**Figure S5.** Time series with trend lines, slope, and *p*-values for the three components (GPP, ET, and T_r_) of WUE at the US-Ne3 site. The red color refers to the first time period (1981–2000), orange refers to second time period (2001–2010), and black represents the complete time period (1981–2010).

**Table S1.** Trends for three definitions of water use efficiency (WUE) from 1981 to 2010 at CN-Qia. The time periods were from 1981 to 2010 (complete period), 1981 to 2000 (first period), and 2001 to 2010 (second period).

|  | **1981–2000** | **2001–2010** | **1981–2010** |
| --- | --- | --- | --- |
|  | **Ecosystem Water Use Efficiency (EWUE)** | | |
| **CLIM** | 0.0012 | 0.0015 | 0.0007* |
| **CO_2_** | 0.0025 | 0.0028* | 0.0030** |
| **AERO** | -0.0001 | -0.0001 | -0.0002 |
| **NDEP** | -0.0009** | -0.0007** | -0.0010** |
| **ALL** | 0.0023* | 0.0027 | 0.0015* |
|  |  |  |  |
|  | **Transpiration Water Use Efficiency (TWUE)** | | |
| **CLIM** | 0.0129 | 0.0331* | 0.0158* |
| **CO_2_** | 0.0150 | 0.0245* | 0.0190** |
| **AERO** | -0.0034 | -0.0040 | -0.0022 |
| **NDEP** | -0.0034** | -0.0066** | -0.0078** |
| **ALL** | 0.0160 | 0.0221* | 0.0153* |
|  |  |  |  |
|  | **Inherent Water Use Efficiency (IWUE)** | | |
| **CLIM** | -2.9081 | -8.0580 | -1.0767 |
| **CO_2_** | 6.8858 | 10.1510* | 5.2281** |
| **AERO** | -1.6918 | -2.2500 | -0.05614 |
| **NDEP** | -2.3600** | -4.1300** | -2.9796** |
| **ALL** | -3.3680 | -12.0440 | -1.4671* |
|  |  |  |  |

* and ** denotes results at 0.05 and 0.01 significance level, respectively.

**Table S2.** Trends for three definitions of water use efficiency (WUE) from 1981 to 2010 at CN-Cng. The time periods were from 1981 to 2010 (complete period), 1981 to 2000 (first period), and 2001 to 2010 (second period).

|  | **1981–2000** | **2001–2010** | **1981–2010** |
| --- | --- | --- | --- |
|  | **Ecosystem Water Use Efficiency (EWUE)** | | |
| **CLIM** | -0.0116 | -0.0292* | -0.0144* |
| **CO_2_** | 0.0019 | 0.0037 | 0.0010 |
| **AERO** | -0.0023 | -0.0039* | -0.0006* |
| **NDEP** | 0.0004 | 0.0003 | 0.0003 |
| **ALL** | -0.0090 | -0.0410* | -0.0182* |
|  |  |  |  |
|  | **Transpiration Water Use Efficiency (TWUE)** | | |
| **CLIM** | -0.0139 | -0.0161* | -0.0114 |
| **CO_2_** | 0.0090 | 0.0043 | 0.0053 |
| **AERO** | -0.0060 | 0.0022 | -0.0034* |
| **NDEP** | -0.0007 | -0.0013 | -0.0009 |
| **ALL** | -0.0142 | -0.0124* | -0.0090* |
|  |  |  |  |
|  | **Inherent Water Use Efficiency (IWUE)** | | |
| **CLIM** | 3.7963 | 6.8120* | 5.9338* |
| **CO_2_** | 1.7702 | 5.4500 | 2.0584 |
| **AERO** | -0.7980 | -2.2040* | -1.4632* |
| **NDEP** | -0.2748 | -0.2700 | -0.5072 |
| **ALL** | 3.6233 | 7.9500* | 5.7349* |
|  |  |  |  |

* and ** denotes results at 0.05 and 0.01 significance level, respectively.

**Table S3.** Trends for three definitions of water use efficiency (WUE) from 1981 to 2010 at US-NE3. The time periods were from 1981 to 2010 (complete period), 1981 to 2000 (first period), and 2001 to 2010 (second period).

|  | **1981–2000** | **2001–2010** | **1981–2010** |
| --- | --- | --- | --- |
|  | **Ecosystem Water Use Efficiency (EWUE)** | | |
| **CLIM** | -0.0033 | -0.0047* | -0.0057* |
| **CO_2_** | 0.0003 | 0.0005* | 0.0008* |
| **AERO** | -0.0009* | -0.0018* | -0.0005* |
| **NDEP** | -0.0006 | -0.0008 | -0.0002 |
| **ALL** | -0.0019* | -0.0063* | -0.0076* |
|  |  |  |  |
|  | **Transpiration Water Use Efficiency (TWUE)** | | |
| **CLIM** | -0.0021 | -0.0035 | -0.0044* |
| **CO_2_** | 0.0032 | 0.0028* | 0.0048* |
| **AERO** | -0.0031* | -0.0016* | -0.0026* |
| **NDEP** | -0.0013 | -0.0011 | -0.0010 |
| **ALL** | -0.0013* | -0.0051* | -0.0064* |
|  |  |  |  |
|  | **Inherent Water Use Efficiency (IWUE)** | | |
| **CLIM** | 4.2049 | 7.9541 | 1.0893* |
| **CO_2_** | 8.2691* | 14.0180 | 6.7265* |
| **AERO** | -2.1557* | -3.5150* | -1.5876* |
| **NDEP** | -1.9808 | -2.4770 | -0.4673 |
| **ALL** | 1.5106* | 8.1458 | 3.0948* |
|  |  |  |  |

* and ** denotes results at 0.05 and 0.01 significance level, respectively.

**Table S4.** Trends for aerosol concentration (AERO) and nitrogen deposition (NDEP) at three study sites during the growing season from 1980 to 2010. The time periods were from 1981 to 2010 (complete period), 1981 to 2000 (first period), and 2001 to 2010 (second period).

|  | **1981**–**2000** | **2001**–**2010** | **1981**–**2010** |
| --- | --- | --- | --- |
|  | **CN-Qia (Evergreen Needleleaf Forest)** | | |
| **AERO** | -0.0001 | -0.0009 | -0.0008 |
| **NDEP** | -0.0021* | -0.0012* | -0.0017* |
|  |  |  |  |
|  | **CN-Cng (Grassland)** | | |
| **AERO** | 0.0003* | 0.0005* | 0.0010* |
| **NDEP** | 0.00002 | 0.00008 | 0.00005 |
|  |  |  |  |
|  | **US-Ne3 (Cropland)** | | |
| **AERO** | 0.0016* | 0.0019* | 0.0023* |
| **NDEP** | 0.0002 | 0.0009 | 0.0007 |
|  |  |  |  |

* Denotes results at 0.05 significance level.
